# “I would stress less if I knew that the nurse is taking care of it”: Multiple Sclerosis inpatients’ and health care professionals’ views of their nursing-experience and nursing consultation in rehabilitation—a qualitative study

**DOI:** 10.1186/s12912-022-01013-x

**Published:** 2022-08-23

**Authors:** Verena Witzig-Brändli, Cordula Lange, Sabine Gschwend, Myrta Kohler

**Affiliations:** 1 Eastern Switzerland University of Applied Sciences, School of Health Sciences, Institute of Applied Nursing Science, Rosenbergstrasse 59, CH-9001 St. Gallen, Switzerland; 2 Rehabilitation Centre Valens, Taminaplatz 1, CH-7317 Valens, St. Gallen Switzerland

**Keywords:** Nursing consultation, Multiple sclerosis, Rehabilitation, Nurse experience, Caring, Qualitative research, Patient perspective, Health care professionals’ team's perspective

## Abstract

**Background:**

Nurses play a crucial role in the multidisciplinary team in the rehabilitation of multiple sclerosis (MS) patients. However, little is known about patients' and health care professionals’ (HCP) experiences (physicians, therapists) with nurses in rehabilitation. The aim of this qualitative study is (i) to describe the rehabilitation nursing care from the perspective of MS patients and HCPs and their view of a nursing consultations (ii) to elaborate similarities and differences of patients’ and HCP’s views.

**Methods:**

We used a qualitative approach and selected the participants purposively. We conducted semi-structured individual MS patient (*n* = 15) and two focus groups interviews with HCPs (*n* = 8) in an inpatient rehabilitation clinic in Switzerland. We analysed the data using a structuring content analysis approach. First, we analysed patients’ and HCPs’ perspectives separately. Afterwards we elaborated similarities and differences descriptively.

**Results:**

Main categories of patients’ perspectives were “need for nursing care” and “relationship between nurses and MS patient”. MS patients have mentioned the following points according to a nursing consultation: (i) nurses as advocates, (ii) involvement of relatives (iii) peer groups (iv) contact person.

“Nurses in their scope of practice”, “nurses as a part of the multidisciplinary team” and “the specifications in the treatment of MS patients” were main categories of HCPs’ perspective.

MS patients and HCPs demonstrated similarly the importance to have a nurse as a contact person in the multidisciplinary team and the need to integrate a nurse-led peer group in a nursing consultation. While HCPs prefer that relative always be included in nursing consultations, patients provided reasons when inclusion was not desirable.

**Conclusion:**

The results indicate that continuity in the nursing care for MS patients could contribute to a trusting nurse-patient relationship. This facilitates nurses to create a deeper understanding of MS patients and their needs in daily rehabilitation. The need for MS patients to share their concerns and receive scientifically proven knowledge from peers could addressed with a nurse-led peer group.

## Background

Multiple sclerosis (MS) is a chronic inflammatory and neurological disease which affects worldwide 2.8 million people [1] with a rising incidence and prevalence [2, 3]. This progressive, incurable disease has an aetiology unexplained fully so far [4]. Due to the typical multifocal lesions in the central nervous system, MS patients suffers from a high symptom load with a variety of symptoms, e.g., fatigue, paraesthesia, spasticity, disfunctions of bladder and bowel, visual problems, depression and emotional lability [4]. MS is often diagnosed at young age, in a progressive manner and the life expectancy of MS patients is steadily rising [5, 6]. MS Patients therefore suffer over a long period from the MS symptoms and their consequences, like a reduced functionality, productivity and negatively influenced health related quality of life [4, 7].

Many of these symptoms and consequences are treated at frequent inpatient intervals by a multidisciplinary team in rehabilitation clinics [8, 9]. The effectiveness of this multidisciplinary rehabilitation approach for MS patients was demonstrated in a Cochrane review. The multidisciplinary rehabilitation not only increased the activity and participation of MS patients, but also improved their quality of life. Patients' disease knowledge increased through interventions that provided information (e.g. consultations) [8].

Rehabilitation nurses are essential members of the multidisciplinary rehabilitation team, their competencies have been recently described by the Association of Rehabilitation Nurses (ARN) [10]. Special role competencies of the rehabilitation nurse are to provide client education (as nursing consultations) and to deliver patient-centred care [11]. This was also confirmed by a Delphi study with Swiss rehabilitation nurses in which patient- and family-centeredness were seen as the highest priority principle for the work of rehabilitation nurses [12]. Further, Gutenbrunner [11] explained rehabilitation nurses as the most important health care professional (HCP) group for building relationships with rehabilitation patients. This prolonged relationship allows rehabilitation nurses to have the best understanding of patients’ contextual and personal factors. This understanding supports the nurses to care for the patients in a patient centered way [11]. In consultations, for example, rehabilitation nurses and patients can work out actions that can be perfectly integrated into the patient's daily routine. However, it is unclear whether these nursing rehabilitation competencies also apply to nurses providing target care to MS patients. Or whether MS nurses still need specific competencies to meet the needs of MS patients.

Little is known about MS nurses in rehabilitation. In several studies, MS nurse interventions had an positive effect on the patients self-management, [13], anxiety reduction [14], quality of life [14, 15] and knowledge of disease management [16–18] but in different settings with in- and outpatients. An integrative review demonstrate that MS nurses are seen as the leading HCP to meet MS patients and carers needs in form of a longitudinal care co-ordinator and a bespoke care provider [19]. This is in line with a UK study, that examined HCPs’ (nurses, therapists, physicians and social workers) and MS patients’ perceptions of a MS nurse [20]. From the MS patients’ view, the care received from MS nurses was experienced through education, rated by 63 percentages (%) of all MS patients, followed by consultations and psychological support (25%). For the HCP, MS nurses primarily meet the MS patient's needs for information about the disease (98%) and for emotional support (91%). HCP and patients consented that MS nurses are the most appropriate professions to provide emotional support [20]. However, little attention has been paid to MS patients and HCP’s view of rehabilitation nurses.

Especially in Switzerland, where MS nurses are still in their infancy, there is a lack of knowledge about the experience of MS nurses in rehabilitation from the view of MS patients and HCPs. Even if MS patients need for nursing consultation has been demonstrated by two Swiss studies [21, 22], no previous study has investigated MS patients and HCPs view of a nursing consultation. Therefore, we aimed (i) to describe the rehabilitation nursing care from the perspective of MS patients and HCPs and their view of a nursing consultation and (ii) to elaborate similarities and differences of patients’ and HCP’s views descriptively.

## Methods

We chose a qualitative descriptive approach to describe the rehabilitation nursing care from the perspective of MS patients and HCP’s. The strength of this approach is the comprehensive description of a phenomenon in everyday terms of those phenomena [23]. It is thus valuable for collecting experiences of patients and HCPs in a contextually relevant way [24]. We reported the results in accordance with the Consolidated criteria for reporting qualitative research (COREQ) [25].

This is a sub study of the MS Nurse project. Its overarching goal is the development, evaluation and implementation of a theory-based nursing consultation for MS patients in rehabilitation guided by the Medical Research Council (MRC) framework [26]. Further information’s are reported elsewhere [27].

### Setting and participants

We conducted this study in one Swiss rehabilitation clinic for neurological disorders, where 327 MS patients are annually cared by a multidisciplinary team of nurses, physicians, and therapists. We used a purposive sampling strategy. One author (S.G.) recruited eligible patients (based on their inclusion and exclusion criteria) from August to October 2020 by screening the medical records. The reasons for using a purposive sampling strategy was to obtain as many cases as possible that are considered information rich for the purpose of this study (e.g. different years of experience as MS patients in rehabilitation, different nursing needs) [23]. We asked 15 MS patients to participate and all of them expressed an interest.

Inclusion criteria for patients were (i) being an inpatient in the rehabilitation clinic, (ii) diagnosed with multiple sclerosis, (iii) and speaking German fluently. Exclusion criteria were (i) severe cognitive deficits measured by the functional independence measure instrument (FIM) [28] and/or (ii) diagnosed with depression.

Inclusion criteria for HCP were (i) member of the multidiscipline team, (ii) speaking German fluently and (iii) had at least one year of work experience in the rehabilitation centre.

### Data collection

We explored the patient’s perspective in 15 individual interviews and the HCP’s view in two focus groups, each with four HCPs. We chose focus group interviews at HCP to determine synergies among HCPs to obtain more in-depth statements on the topic of the nurse's experience on the team. Similar and different perspectives of individual HCPs were identified in the focus group and could be discussed collectively [29]. Whereas the individual interviews focused on the patient’s unique experience with nurses from perspective of MS patients [30]. The research team developed two semi-structured interview guides, one for the patients’ interviews and one for the interviews with the HCPs [31] based on literature [10, 12, 19, 20] and the authors’ experience (S.G. and M.K.) in the field. Two authors (S.G. and M.K.) of this study have been working in this rehabilitation clinic for several years. Follow-up questions were added to the guides based on their experience with the needs of people with MS. The guides included major topics as “nursing care experience” “expectations and needs of nursing care” (patients), “experience of day to day work with nurses” and “expectations of a nursing consultation” (HCP). Two authors (S.G. and M.K.) conducted the focus group interviews (one each) and a research assistant took field notes. We used this field notes as memos especially as help during transcription and analysis process. One author (S.G.) conducted patient individual interviews. New topics which arose during the patient interviews were added to the guide.

The interviews took place in a separate, quiet room of the rehabilitation clinic. They lasted between 11 and 45 min (mean = 26) for patients and 47 to 60 min for focus groups interviews (mean = 53). We audiotaped all interviews and we transcribed them verbatim. We pseudonymized all the patients’ and HCPs’ personal data.

### Data analysis

This qualitative study used a structuring content analysis close to Mayring [32]. The data analysis is shown on the basis of the seven steps of the toolbox of Schreier [33]. First, after defining the research questions, we selected the transcripts sections which answered the research questions. One author (V.W.B.) analysed the patient and one other author (C.L) the focus groups interviews using the software MAXQDA 2020 [34]. To create a category system, we decided to use a deductive-inductive approach based on the interview guide. We read the transcripts several times and then performed open coding. For the subdivision of the transcribed-material into topic clusters, we built inductive codes into primary categories. We added these categories to the deductive categories of our interview guide and complemented them. We performed a trail coding with one patient and one focus group interview and discussed it with the last author (M.K.). We analysed the patients and HCPs data independently. In the analytic process, we discussed the analyses with the co-authors (S.G. and M.K.) several times.

In a next step, we compared the two analyses of the different perspective (patients and HCP) descriptively. Two authors (V.W.B and C. L.) discussed similarities and differences of the perspectives and illustrated them into two tables.

When presenting quotes in the results section, we use square brackets to note additional information for comprehension. The brackets at the end of the quote indicate the interview number, followed by the corresponding line in the transcription.

### Rigour

According to the concept of trustworthiness of Guba and Lincoln credibility, dependability, transferability and confirmability are criteria for qualitative research [35]. To enhance credibility in the data collection process, one author (S.G.) with a prolonged engagement in the rehabilitation clinic conducted the patient interviews based on the interview guide. Continuous debriefings with the last author (M.K.) served as a reflection on the collection process. Interviewer triangulation during the focus group interviews also enhanced credibility. The analysis was performed by two authors (V.W.B. and C.L.), who did not work in the rehabilitation clinic. They repeatedly discussed the findings with other authors (S.G. and M.K.) to achieve the consistency between researcher’s analysis presentation and the clinical rehabilitation setting. As the analysis was in German, a bilingual speaker of German and English checked the translated quotes. To achieve transferability, we described the characteristics of the participating patients and the setting. We ensured confirmability by considering field notes and verbatim transcripts. In a confirmability audit, we discussed the analysis several times with our research colleagues, who were not involved in the topis of rehabilitation nursing care. Additionally, we used quotes to illustrate the findings.

## Results

A total of fifteen MS patients and eight HCPs (nurses = 3; physicians *n* = 2; physio-occupational therapist, psychologist each *n* = 1) were interviewed. No participant dropped out. Demographic and clinical information of the patients are listed in Table 1. Patients’ mean age was 55 years and they were diagnosed with MS on average nine years ago. Table 2 provides information per interviewed patient. HCPs’ mean work experience with MS patients was 13 years and their mean length of working in the setting was 10 years.

**Table 1:** Patients’ characteristics

(*n* = 15)	
Age in years mean	55
Gender female n (%)	9 (60)
Diagnosed since in years mean	19
FIM mean	78
EDSS mean	6.5

*Abbreviations*: *EDSS* Expanded Disability Status Score, *FIM* Functional independence measure

Expanded disability status score is a method of quantifying disability in MS. The scale (based on an neurological examination) measures the impairment of eight functional systems as cerebral-, visual- or pyramidal functions. EDSS ranges from zero (= normal neurological exam, no disability) to ten (= death due to MS) in 0.5 unit increments. EDSS 1.0 to 4.5 refer to MS patient who are able to walk without any aids. EDSS steps 5.0 to 9.5 are defined by MS patients unable to walk [36]

Functional independence measure is an 18-item scale assessing six areas of functions (self-care, transfer, communication, …). Each item is scored on a 7-point Likert scale. The higher the score, is for an item, the more independent is a MS patient at preforming the item ( 1 = total assistance, 7 = total independence). The items fall into two domains, which are referred to the motor- and cognitive FIM. A total FIM score ranges between 18 to 126 [37, 38]

**Table 2:** Patient characteristics per interview

Number of the interview	Gender	Age in years^a^	MS Diagnoses since x in years^b^	FIM at entry to rehab	EDSS
1	Men	30	5	106	5
2	Female	40	5	102	5
3	Female	60	5	105	5.5
4	Female	60	30	114	5
5	Men	40	20	51	7
6	Men	60	15	102	4
7	Female	60	30	34	9
8	Men	60	25	63	6.5
9	Female	60	15	69	7
10	Men	60	30	106	6
11	Female	60	25	38	8
12	Female	50	20	79	6.5
13	Men	60	25	48	8.5
14	Female	70	20	58	8
15	Female	60	15	95	6.5

*Abbreviations*: *EDSS* Expanded Disability Status Score, *FIM* Functional independence measure, *rehab* rehabilitation

^a^rounded to steps of 10

^b^rounded to steps of 5

### MS patients’ description of rehabilitation nursing care

In the part of the patients’ description of rehabilitation nursing care the analysis revealed two main categories “need for nursing care” and “relationship between nurses and MS patients”.

Needs for nursing care: Patients described situations in which they needed nursing care and situations in which there was no need. No need of nursing care was indicated when patients were independent in bathing, showering, toilet hygiene and mobility.

I am relatively independent. That means I can go to the toilet by myself, eat by myself, I can shower by myself. (06 / 30)

These patients had contact with nurses for short instances, for example to measure vital signs. They did not have a deep relationship with the nurses and could not imagine the tasks of the nurses.

I don't need nursing care because I take my medication on my own. I don't need any support. I only have contact with them [nurses] when they measure my blood pressure. If I have a question, I ask the physician or the therapist. (03 / 22)

These patients focus on the rehabilitation clinic was to receive sufficient therapies. Patients with a higher need for care, had more often and longer contact with nurses and were able to describe the nurses’ tasks more accurately.

I had received a urinary catheter in the hospital. At the beginning, I had a lot of problems with it. I felt very insecure. The nurses kept showing me why I needed this urinary catheter and supported me in the handling. (14 / 12)

The relationship between nurses and MS patients: Relationships were described by patients to be an essential part for a better collaboration between nurses and patients.

It also needs the social exchange. In the evening, when I get assistance to go to bed. Then she (the nurse) should not only cover my legs, but also talk with me a little. (14 / 8)

A beneficial factor for building a relationship is the continuity in nursing care. Continuity in nursing care was defined by patients as being taken cared by the same nurse every day.

It is important to me that the same person [nurse] always assists me in the morning when I take a shower. When a new nurse arrives, I always have to explain from the beginning how she has to assist me. This is frustrating. The nurse must know me personally and my conditions. That's the only way we can work as a team. (05 / 36)

Through continuity and a trusting (long-term) relationship, nursing care was experienced in a more holistic way. This allowed patients to talk more openly about their personal concerns, e.g. fear of the future or having to depend on relatives. Moreover, continuity in nursing care was an essential factor in ensuring patients that nurses did not lose medical patient information, for example how to manage high blood pressure.

I have problems with high blood pressure. When the same nurse comes every day, she already knows what time I take the medication and when the blood pressure should be measured. When another nurse is responsible for me, the times are no longer correct. Then I have to remind the nurses to measure my blood pressure on time. (15 / 93)

Prerequisite for a deep relationship were nurses’ personal characteristics. Nurses were expected to adopt a caring attitude, so they had to be willing to respond to the patients’ needs and have a respectful attitude, as one patient described:

You never have the feeling you are being a nuisance. (03 / 38)

Another facilitator for a trustworthy relationship is knowing each other for a long time. Some patients had been regular visitors of the rehabilitation center for years.

I know a lot of people [nurses, therapist, physicians] here. I saw them when I was here again and again. I am very happy when no new nurses show up and I do not need to get to know them again. (05 / 4)

Knowing each other for such a long time, living a trusty relationship results in everyday situations in which nurses and patients understand each other without words. One example is the morning washing routine. If the nurse knows their patients, their needs and preferences in depth and respects them, explanations form the patient during the morning routines are no longer necessary.

I like it when the same nurse comes every day. She knows my morning routines and she doesn't have to ask everything again. I don't have to explain everything again. Because she already knows what my preferences are and where I need support. (07/ 42)

### MS patients’ view of a nursing consultation

MS patients view of a nursing consultation includes four main categories “MS nurse as an advocate”, “the involvement of relatives in the consultation”, “the need of a peer group” and “the need of a contact person”. In the following section, the nurse who provides nursing consultation is referred to as the MS nurse.

MS Nurse as an advocate: Trusting the MS nurse to act on their own needs was described as particularly valuable and was seen as a great relief for patients. For some patients, part of having a trusting relationship with a MS nurse, means opening up a little more by showing more emotion. The idea that a MS nurse is constantly performing a consultation would cause patients to open up even more.

So that I could be completely honest about my emotions and say that today I am really feeling like shit, and it would be nice if someone [MS nurse] had time to listen to me and maybe comfort me a little bit. (15 / 50)

In addition, patients also described imagining that the MS nurse would act as an advocate. The nurse should advocate for the patient’s wishes and needs in the multidisciplinary team.

Now, I am suffering from fatigue. I would share this with the MS nurse. She could then discuss with the therapists if we can schedule the therapy sessions to be less exhausting. (02 / 52)

The involvement of relatives in the consultation: Patients also wished their relatives to be their advocates. Patients believed that if relatives could join a nursing consultation, they would have a better understanding of the situation and their illness. In addition, patients saw the nursing consulting as a place where they could talk with their relatives about issues they had not discussed before but were important to them. Possible topics included changing sexuality or loss of independence.

It is not nice when you see your partner becoming fragile. After all, you have been with that person for 20 years when he is diagnosed. Hey, it is not like the ground collapses, it is like the whole world collapses. (08 / 64)

Even though it was a major need of the patients to involve relatives, patients also described some challenges. Several relatives did not want to be more involved.

He just does not want to hear it. He always says: "I live with you, I know enough.” (12 / 126)

Some patients did not want that their relatives to get involved. In their opinion there was no need for relatives to be part of the nursing consultation, as long as they had another HCP who was confident such as the General practitioner (GP) or a neurologist.

If there are problems with my disease [MS], then my girlfriend can always contact the GP. She does not need an extra person to talk about the disease. (06 / 48)

The need of peer groups: To share concerns and to learn from peers was seen as another need for a nursing consultation. A few patients emphasised the importance of a guided peer group by a nurse. In this way, they want to be guaranteed that the knowledge they have acquired is scientifically proven.

I want to exchange information with other MS patients in the nursing consultation and then I want her [MS nurse] to confirm whether the information exchanged is correct or not. (01 / 49)

Some patients already had experience with a peer group and mentioned many positive aspects.

Then you also have an exchange with each other, and you get an understanding of the disease, and you can share your know-how. (15 / 44)

The need of a contact person: Patients stated that without a MS nurse they had difficulty directing their questions about disease and symptom management to the right person during their inpatient rehabilitation. It was unclear to them who, of the multidisciplinary team, was in charge for which topic. Patients described the need to know a defined contact person, such as a MS nurse in the multidisciplinary team.

That I do not have to run from one person to the next person (...) that I can safely forward my question (…) I would stress less if I knew: "she's [MS nurse] taking care of it". (12 / 108)

Especially newly diagnosed, less rehabilitation-experienced patients, with less needs for nursing care were more concerned to ask the wrong person in the multidisciplinary team.

I realize that every now and then I have questions that I might raise to the wrong person. (06 / 14)

In contrast, patients with a long illness history, with many years of rehabilitation experience and patients who needed a lot of nursing care, developed strategies of choosing the right contact person. One patient described his strategy for selecting a contact person for disease-related issues as follows.

So already with the nurse, or with a therapist, or if it is more complicated, then I go to the doctor. (04 / 38)

After discharged from the rehabilitation clinic, the problem of a missing contact person is intensified. Patients report, that in many settings (e.g. outpatient nursing services) MS specific knowledge is rare.

I would be grateful if I could just call her [MS nurse] and make an appointment. Because in XX [hometown] I would not know who to ask. If I imagine when I need more assistance with nursing care, then I would appreciate knowing where to get the information. (04 / 52)

As such a contact person is currently missing MS patients were struggling to find their needed information on other sources, as the internet or peers. They are often exhausted from searching. MS patients become frustrated when they do not get their information and then stagnate in their search even if they know how important the information is. Knowing who to contact with questions regarding the disease management after rehabilitation is a major patient need.

### HCPs’ description of rehabilitation nursing care

In the focus groups interviews the three main categories are included “the nurses in their own scope of practice”, “nurses as a part of the multidisciplinary team” and “the specifications in the treatment of MS patients”.

Nurses in their own scope of practice*:* Having a deep insight into patients’ daily lives was considered as a typical nursing characteristic. Due to their 24/7 presence, they often had more and longer contact to patients than other HCPs. Nurses described that MS patients reported more personal topics in conversations in between or after therapy sessions (night-time), especially if a relationship of trust already existed. This extended presence of the nurses had also been noticed by the physicians.

Because the other disciplines only see the patient selectively and then the nurses see them in more detail or longer. They have an additional time aspect that we [physicians] lack. (FG 2 / 17)

Some of the topics raised would have never been discussed in therapy sessions or during medical visits. Nurses could therefore actively address important issues with patients. They had extensive knowledge about patients compared to other team members. Therefore, nurses were seen as a source of patient information for the team. Additionally, nurses became the voice of patients to advocate for their patients’ needs in the team.

Currently, the role of nursing is also being a spokesperson and a supporter, a patient advocate. (FG 1 / 14)

Nurses as a part of the multidisciplinary team: In both focus groups interviews the individual team members saw themselves as one team, whose members discussed and determined patient goals. They saw the patients’ needs as the basis of all their actions.

Our actions must therefore be guided by the needs of our patients. (FG 1/45)

Nurses were important members of the team. HCPs recognised that nurses’ positions in the team sometimes were not visible. One example was given that nurses did a poor job of representing their point of view during multidisciplinary visits. Also, nurses agreed that they were often not heard in the team.

Sometimes I have the feeling that we are not being heard (…) Or still not recognised. That is certainly a barrier (…). (FG 2/45)

Specifications in the treatment of MS patients*:* HCPs agreed that MS patients are more likely to have recurrent stays compared to other rehabilitation patients. Some MS patients have been recurrent rehabilitation patients for years. As a result, HCPs and MS patients knew each other well.

The nurse already knows the patients very well. For example, if a patient is in the same ward for the sixth time, the nurse already knows very well what his preferences and wishes. (FG 01 /16)

### Similarities and differences of the MS patients’ and HCPs’ perspective

MS patients’ and HCPs’ experience of the nursing care were compared descriptively. Similarities and differences are listed in Table 3.

**Table 3:** Patients’ and HCPs’ experience of the nursing care, their similarities, and differences

Patients’ perspective	Similarities between patients and HCPs'	HCPs’ perspective
	**Need for nursing care (independent patient)**	
Consider oneself as not in need for nursing care	Same definition: No need for nursing care was described in case of patients who are independent in physical activities like toilet hygiene. The focus on the rehabilitation of these patients is on therapies	Regardless patients independence, all MS patients demonstrate needs for nursing care (e.g. being available and present for patients).
	**The nurse as contact person**	
Targeted selection of contact persons by patients with long illness histories, many years of rehabilitation experience and need a lot of nursing services	Same difficulties: To identify a contact person. Different disciplines have primary contact persons	Choice of a contact person depends on situational factors and already existing trusty relationship
Unspecific selection of contact persons by patients with new diagnose, independent in nursing services, less rehabilitation experience		Unspecific selection of contact person by: patients with many years of rehabilitation experience
	**Definition, advantages and disadvantages of a continuous nursing care**	
Supplement advantages: Nursing care is more tailored to the needs of MS patients	Same definition of continuous care: To be cared for by the same nurse every day. This includes same required competencies in in-depth MS knowledge Same advantages of continuity: No loss of information	Supplement definition: To be available 24/7 Disadvantages**:** HCPs suffer from demanding patients

*Abbreviations*: *24/7* Be available 24 h at seven days, *HCP* Health care professionals

Both groups agreed that it was difficult for patients to find a contact person in the multidisciplinary team. The strategies on how to choose such a person differed in the HCPs’ and the MS patients’ perspectives. MS patients and HCP agreed that trust and continuity are important factors for the nursing care. But HCP also realized some limits of the continuity. The HCPs reported that MS patients could be demanding. The complexity of the physical and psychological symptoms of MS patients was a challenge for nurses. Caring for these patients over an extended period could be very stressful for a single nurse. Nurses described that it was helpful to hand over responsibility for a patient to a nursing colleague for a short time to gain distance from the situation. This distance enabled nurses to fully re-engage with the patients.

The view of the patients and HCPs on a nursing consultation is listed in Table 4. Both agreed that a nurse who performs these consultations, needed MS knowledge and socials skills such as trustworthiness. While for the HCPs a key point was to “think multidisciplinary”, the patients desired that the nurse act as an advocate for their needs in the multidisciplinary team. The HCPs prefer that relatives were always involved in consultations, while patients commented reasons why an involvement was not desirable.

**Table 4:** Patients’ and HCPs’ perspectives of a nursing consultation intervention, their similarities and differences

Patients’ perspective	Similarities between patients and HCPs	HCPs’ perspective
	**Requirements for the MS nurse offering the service**	
MS nurse as an advocate	Same need for a continuous contact person: MS nurse need competence to have a trustful relationship with MS patients	Working multidisciplinary
	**Involvement of the relatives**	
Situations, when relatives should not be part of the nursing consultation intervention	Relatives have to be a part of the service	Relatives should always be part of the nursing consulting
	**The need of a peer group**	
Challenges: To know how valid shared information is	Same Advantages: To learn from each other	Challenges: To recruit enough suitable peers
Match peer with same MS type or with same timeline in illness history	To choose peers carefully	Caution: Not to overwhelm new diagnosed patients when they meet seriously ill patients
		Peer groups need a leader and a predefined topic

*Abbreviations*: *GP* General practitioner, *HCP* Health care professionals

## Discussion

These study findings revealed aspects of nursing care and nursing consultations from the perspective of MS patients and HCP’s in one Swiss rehabilitation clinic. MS Patients described nursing care according to the categories “need for nursing care” and “the relationship between nurses and MS patients”. In the HCPs perspective, rehabilitation nurses are part of the multidisciplinary team. In addition, HCPs indicated that nurses define themselves by their own scope of practice, in which the treatment of MS patients is a special area of rehabilitation nursing. MS patients named the following important points according to a nursing consultation: (i) nurses as advocates, (ii) involvement of relatives (iii) peer groups (iv) contact person. The last two category were identical with the HCP needs. While HCPs prefer that relative always be included in a nursing consultation, patients provided reasons when inclusion was not desirable.

### Relationship between MS patients and nurses

Having emotional support through a trusting and continuing relationship with a nurse was a need of many MS patients. To choose a nurse as a confident person matches with the results of Gutenbrunner et al. (2001), where nurses have the most continuous relationship to rehabilitation patients and have therefore the best insight into patients’ daily life and in their context [11]. This will allow the nurses to have a great impact on patients’ care and their health outcomes. A meta-analysis showed that a trusting relationship lead to better health behaviours, better quality of life and more satisfaction with the treatment in patients suffering from chronic or multiple health complaints [39]. Also, MS patients benefited from a collaborative partnership with a MS nurse, as the nurses increased patients’ understanding of the illness and the treatment options and encouraged their adherence [13]. The importance of a trusting relationship between MS patients and nurses was also reported by While et al. stating that MS nurses are key providers for emotional support [20].

In contrast, another Swiss study showed that relational caring in a relationship of inpatients and nurses was rated as the least important by elderly rehabilitation patients. Inpatients did not desire to live a deep relationship to nurses [40]. This discrepancy may be due to the different population, as MS patients are more likely to have recurrent rehabilitation stays. We assume that this fact will increase the importance of a relationship. The importance of a deep relationship with a nurse as a confidant is also confirmed by the ARN clinical practice guidelines. These guidelines recommend that nurses serve as advocates for patients and their families to support them in the access to care. Continuous and sustained care is recommended along the full spectrum of MS care [41].

### Nurse as a contact person

To know a nurse as a contact person to meet unmet needs is seen by patients and HCPs as essential. Our study is in line with a previous UK study, where they showed that two years after the first appointment with a MS specialist nurse, significantly more participants could name an available contact person than when they were treated with usual care (appointments with physicians, therapists) (*p* = 0.01). Further, MS patients in the intervention group suffered from significantly less pressure ulcers than MS patients of the control group (*p* < 0.001) [18]. To know where to ask questions about MS specific symptoms was a need of several MS patients. Similarly, a Swiss cross-sectional study with MS patients and their relatives examined that needs for information about the disease are the most unmet needs followed by counselling needs [21]. Nevertheless, not all interviewed patients did have a need for a contact person. Some already had other HCPs as contact persons.

In an HCP perspective not only to know the patient, but also to know their relatives was a major topic. Previous studies have already reported that family involvement was important for MS patients [42, 43] and their relatives [44, 45]. Similarly, in the competency model for professional rehabilitation nursing developed by the ARN patients are seen in the system of their families. Relatives should therefore use the support structure of the patients equally [10]. Our results highlighted the importance of staying in direct contact with the relatives because the motivations for and against participation differed between patients and relatives.

### The need to integrate a nurse-led peer group in a nursing consultation

The need of a nurse-led peer group was mentioned by the several MS patients. This need is matched by a Finish study in which 68 MS patients cited peer group support as one of seven major rehabilitation components [46]. Peer groups of MS patients have a positive effect of the self-transcendence, the physical health score (*p* = 0.001) [47], the problem-oriented coping strategies [48] and the quality of life [49] compared to patients without a peer-group support. In our study, MS patients and HCPs stated, the formation of the peer group should be well thought through, as MS patient do have different needs for a peer group. This was demonstrated in an Australian study, in which female patients with several symptoms needed more varieties in the peer groups than male patients [50].

### Strengths and limitations

The strength of this study is the two perspectives on the nursing experience in one rehabilitation clinic. At a methodological level, a purposive sample allowed us to reach a broad range of MS patients with different rehabilitation nursing experiences [23]. Due to the limited number of MS patients in a rehabilitation clinic, this purposive sampling approach supported us to ensure that several MS patients had the opportunity to participate in our study in a short period of time. We wanted to give every MS patient in the rehabilitation clinic the chance to participate in the study. With a screening of medical records, we were able to reduce the risk of missing a MS patient during the recruitment phase, who stayed as inpatient in the rehabilitation clinic.

Another strength of this study is the choice of individual MS patients, and the focus group interviews. While a better collectively understanding of the nurse experience was generated in the focus group, patients felt freer to talk about intimate topics (eg. shower assistance) in the individual interviews. We assume that patients in a focus group would have talked less about such intimacy issues. To be transparent in the comparison of data of two different interview types, we have only conducted a descriptive comparison.

One author (S.G.) as a clinical nurse specialist conducted the interviews with the patients’, which could lead to social desirability bias [51]. Patients could project a positive image of themselves to the interviewer to generate a positive image or to remain in a good relationship with the author. Both issues might decrease MS patients’ willingness to discuss specific issues, which could be a bias for the data. Our strategies to reduce this bias were regular debriefings between the interviewer and with the last author. However this close relationship between MS patient and interviewer is also noticed as a strength. Since the interviewer was already known by the patients, it might have been easier for them to open up about the intimate topics mentioned above. Another limitation could be that the interview guidelines were not piloted, but this is opposed by the very limited time resources of the patients and the treatment team. Even though the analysis conducted by two authors who do not work in a rehabilitation setting, they were also close to the topic because of their professional nursing background. These backgrounds could influence our results. We therefore discussed the analysis with non-nurse research colleagues. Nevertheless, MS patients and HCP’s provided rich data in the German-speaking rehabilitation context.

## Conclusion

This study gives an overview of the patients’ and HCPs' perspective of the current MS nursing care in the rehabilitation and the resulting needs regarding a consultation nursing intervention. Key findings indicate MS patients need of a continuous nurse relationship. While HCPs emphasized the advantages of a continuous relationship between nurses and MS patients, they also mentioned its limitations (e.g. demanding aspects of caring).

Our results indicate that continuity of planning by the same nurse for the same MS patient in rehabilitation is crucial. Through this continuity, a trusting relationship can be established. This relationship facilitates nurses to create a deeper understanding of the MS patients and their needs in daily rehabilitation routines. The results of this study also suggest that, especially during the planning of a MS nursing consultation, relationship-related aspects might be important to meet MS patients’ needs. The need for MS patients to share their concerns and receive scientifically proven knowledge from peers could addressed with a nurse-led peer group. Further work is required to establish the unmet needs of the relatives of MS patients in the rehabilitation clinic.

## Data Availability

The datasets generated and analysed during the current study are not publicly available due to patient privacy and ethical restrictions but are available from the corresponding author on reasonable request.
